# The TGFβ Family in Human Placental Development at the Fetal-Maternal Interface

**DOI:** 10.3390/biom10030453

**Published:** 2020-03-13

**Authors:** Susana M. Chuva de Sousa Lopes, Marta S. Alexdottir, Gudrun Valdimarsdottir

**Affiliations:** 1 Dept. Anatomy and Embryology, Leiden University Medical Center, 2300 Leiden, The Netherlands; S.M.Chuva_de_Sousa_Lopes@lumc.nl; 2 Dept. Reproductive Medicine Anatomy and Embryology, Ghent University Hospital, 9000 Ghent, Belgium; 3 Department of Anatomy, BioMedical Center, University of Iceland, Sturlugata 8, 101 Reykjavik, Iceland; msa8@hi.is

**Keywords:** human placental development, fetal–maternal interface, epithelial-to-mesenchymal transition of trophoblasts, mesenchymal-to-endothelial transition of extravillous trophoblasts, TGFβ family, preeclampsia, human trophoblast stem cells, organoids

## Abstract

Emerging data suggest that a trophoblast stem cell (TSC) population exists in the early human placenta. However, in vitro stem cell culture models are still in development and it remains under debate how well they reflect primary trophoblast (TB) cells. The absence of robust protocols to generate TSCs from humans has resulted in limited knowledge of the molecular mechanisms that regulate human placental development and TB lineage specification when compared to other human embryonic stem cells (hESCs). As placentation in mouse and human differ considerably, it is only with the development of human-based disease models using TSCs that we will be able to understand the various diseases caused by abnormal placentation in humans, such as preeclampsia. In this review, we summarize the knowledge on normal human placental development, the placental disease preeclampsia, and current stem cell model systems used to mimic TB differentiation. A special focus is given to the transforming growth factor-beta (TGFβ) family as it has been shown that the TGFβ family has an important role in human placental development and disease.

## 1. Human Fetal-Maternal Interface

### 1.1. Placental Development

**Figure 1:**
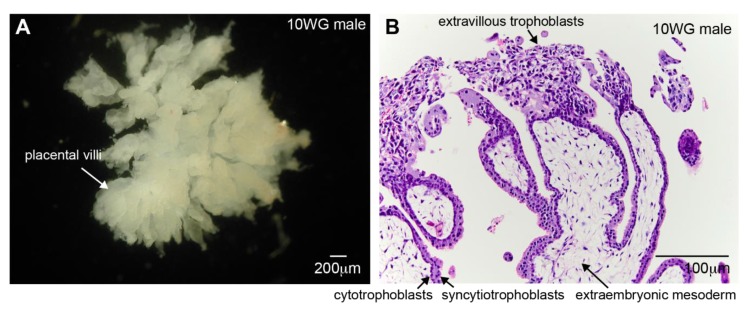
Structure of human placental villi early during pregnancy. (**A**) Photograph of part of the placenta that interfaces with the uterus (top view) containing the tops of the placental villi at 10WG. (**B**) Histological section of placental villi showing the core of extraembryonic mesoderm and the outer layers of cytotrophoblasts (CTBs), surrounded by syncytiotrophoblasts (STBs). The top of the villi contains a column of CTBs, and the extravillous trophoblasts (EVTs) will invade the uterus. The section shows an eosin and hematoxylin staining. Scale bars are 200 micrometers in (**A**) and 100 micrometers in (**B**).

During pregnancy, the placenta is the first temporary endocrine organ to develop from cells originating from the embryo [1]. The placenta produces several steroid hormones, including estrogen and progesterone, which are pivotal in the maintenance of healthy pregnancy [2]. The main role of the placenta is to anchor the conceptus; prevent maternal immune responses; and provide an interface to exchange nutrients, oxygen, and waste products [3]. During embryonic development, the embryo undergoes its first differentiation when inner blastomeres differentiate into the inner cell mass that later forms the embryo proper as well as extraembryonic mesoderm, whereas the outer blastomeres differentiate into the trophectoderm that gives rise to the extraembryonic trophoblasts (TBs). When the blastocyst is implanting and is only partially embedded in the maternal endometrium, the TBs further differentiate into cytotrophoblasts (CTBs) and hyper-invasive syncytiotrophoblasts (STBs) that form lacunae. Both cell types will give rise to the placental villi: the STBs form the outer layer (multinucleated epithelial syncytium) that is in direct contact with the maternal blood in the intervillous space, and the CTBs form a continuous cuboidal layer just beneath the STBs (Figure 1). The core of the placental villi will be gradually filled with extraembryonic mesoderm that will become vascularized with embryonic blood vessels. At the contact with the maternal uterus, CTBs differentiate into invasive extravillous trophoblasts (EVTs). Further growth and development of the TBs and extraembryonic mesoderm leads to branching of the villi, wherein embryonic mesenchymal cells develop into primitive blood vessels in a process of vasculogenesis [3].

### 1.2. The Uterus During Human Development

During reproductive life, the uterus is a dynamic organ that undergoes dramatic monthly cyclic remodeling (proliferative, secretory, menstrual phase) in response to cyclic changes in the levels of progesterone and estrogen [4]. The uterus is composed of three layers: the outer perimetrium, the muscular myometrium that harbors the plexus vascularis, and the inner endometrium. The endometrium is a hormonal reactive tissue formed by two tissue layers. The tissue layer in contact with the myometrium, the basal layer, contains the base of the uterine glands, where their stem cells reside. This layer is not eroded during menstruation, and reconstructs the structure of the endometrium after the menstrual phase. The most inner layer of the endometrium in contact with the uterine cavity, the functional layer, is rebuilt every reproductive cycle and shed during menstruation. The endometrium is lined by a layer of luminal epithelium that is continuous with the (tubular) uterine glands, and under the luminal epithelium is the mesenchymal stroma (or connective tissue) harboring the spiral arteries. Spiral arteries are specific to the uterus and play important functions. They are able to extend due to the spiral morphology as the endometrium layer grows in thickness from 1 to 5 mm in preparation for pregnancy (secretory phase). In the absence of a pregnancy, the spiral arteries contract, resulting in hypoxia of the functional layer and this tissue layer sloughs off (menstruation). Thereafter, it is important that the (spiral) arteries close to allow reconstruction of the endometrium during the next menstrual cycle [5].

**Figure 2:**
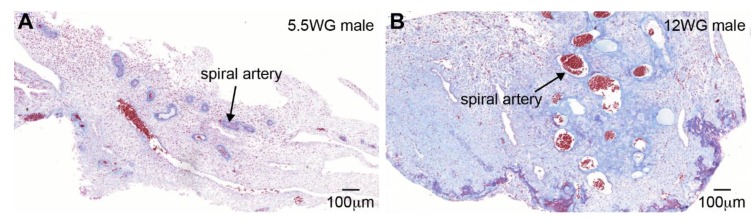
Spiral arteries in the human maternal uterus. Histological sections of decidualized endometrium showing unremodelled spiral arteries during early pregnancy at 5 weeks and 5 days of gestation (**A**) and remodelled spiral arteries later in gestation at 12 weeks of gestation (WG) (**B**). Sections show AZAN (azocarmine G, phosphor-tungstic acid, orange G and aniline blue) staining. Scale bars are 100 micrometers.

In case of a pregnancy, about 1 week after ovulation, the endometrium in late secretory phase is ready to accommodate the embryo and undergoes decidualization, a specialized process whereby the mesenchymal stroma transforms into decidual stroma. Decidual stroma is characterized by its edematous structure, extracellular remodelling, and strong infiltration of immune cells, in particular uterine natural killer cells, which will play an important role in the remodeling of the spiral arteries [6]. During normal placental development, the uterine spiral arteries undergo extensive remodeling of high to low resistance, thereby providing the placenta with adequate perfusion [7] (Figure 2). During decidualization and the first trimester of pregnancy, the uterine glands also remodel extensively and are the source of nutrients (“uterine milk”), including glycogen, to the developing embryo, as the connection with the maternal arterial circulation (spiral arteries) is only established around 10 weeks of gestation (WG) [8].

## 2. Trophoblast Invasion

Implantation and placentation are species-specific processes, and this adds to the difficulty of studying them and their associated pathologies [9]. The depth of trophoblast invasion and spiral artery remodelling mechanism (endovascular route and interstitial route) seems common between great apes (human, chimpanzee, gorilla), but differs from gibbons and baboons, which show shallower endometrial invasion and exclusive use of the endovascular route [10,11,12].

**Figure 3:**
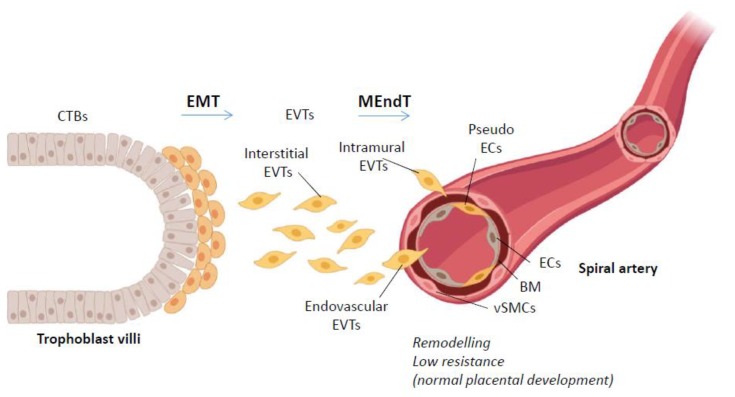
Epithelial-to-mesenchymal transition (EMT) and mesenchymal-to-endothelial transition (MEndT) in human placental development at the fetal–maternal interface. When CTBs differentiate in EVTs, they undergo EMT and invade the decidua. E-cadherin expression is decreased and EMT transcription factors are upregulated. Matrix metalloproteases (MMPs) and lysyl oxidases also need to be activated. Then, the EVTs (both endovascular and intramural) undergo MEndT during the arterial remodelling of the maternal spiral artery. The expression profile of these pseudo-ECs is characterized by upregulation of the vascular markers vascular endothelial (VE)-cadherin, platelet endothelial adhesion molecule-1 (PECAM-1; CD31), vascular cell adhesion molecule-1 (VCAM-1), α4 integrin, and αvβ3 integrin.

During the first trimester, the CTBs at the tip of the anchoring placental villi that make contact with the material endometrium differentiate into EVTs. The invading EVTs can be subdivided into interstitial EVTs that migrate and invade the decidual stroma, epithelial EVTs that line the maternal decidua (together with maternal cells), endovascular EVTs that invade the maternal spiral arteries replacing the endothelial cells (ECs), and intramural EVTs that remove the vascular smooth muscle cells (VSMCs) from the tunica media in the endometrium and sometimes even in part of the myometrium [7,13] in a process that is often termed pseudovasculogenesis (Figure 3). Recently, the single-cell transcriptome profiles of the fetal–maternal interface from 6-14 WG (both decidua and placenta) revealed the molecular signature of the different cell types important to understand placentation [14].

### 2.1. EMT in Trophoblast Invasion

When CTBs differentiate in EVTs, they undergo epithelial-to-mesenchymal transition (EMT) and thereafter they go through a process of mesenchymal-to-endothelial transition (MEndT) during arterial remodelling (Figure 3). EMT is a process in which epithelial cells lose their polarity and adhesion, changing to a mesenchymal-like phenotype by the gain of migratory behavior [15]. In general, the EMT transcription factors such as SLUG, SNAIL, and TWIST become upregulated and in turn the expression profile of the cell changes from the loss of E-cadherin to the gain of N-cadherin and vimentin [16]. These EMT factors have also been shown to play an important role in human TB invasion [17]. However, many known EMT factors in various cell types have not been investigated in the transition from CTBs to EVTs. During invasion, the EVTs upregulate matrix metalloproteases (MMPs) such as MMP2, MMP3, and MMP9 that degrade the extracellular matrix (ECM) [18,19].

Lysyl oxidases (LOX) are a family of extracellular copper-dependent enzymes that catalyze the cross-linking of collagen and elastin in the ECM by oxidative deamination. This makes the proteins insoluble and thus maintains the structural stability and stiffness of the ECM [20]. Besides the known function of ECM cross-linking after secretion of lysyl oxidases, emerging data elucidate their roles intracellularly [21]. The LOX family contains five members: LOX and LOX-like (LOXL) 1, 2, 3, and 4. The function of LOXs is both required and strictly controlled during normal development. However, unusual expression and activity of these proteins has been linked to diseases such as cancer [22] and placental development [23].

### 2.2. MEndT in Trophoblast Pseudovasculogenesis

Endothelial cells can undergo endothelial-to-mesenchymal transition (EndMT) when adopting cardiac fibroblast-like phenotype and vice versa MEndT during acute ischaemic cardiac injury [24,25]. Ubil and colleagues demonstrated that upon starvation, MEndT was mediated through increased expression of the transcription regulator p53, a well-known player in regulation of DNA repair, cell cycle, and apoptosis [25]. Pseudovasculogenesis could indeed be described as MEndT, where migratory EVTs convert their phenotype to endothelial-like cells (Figure 3). The remodelling of the spiral arteries takes place in the endometrium and can even reach the first third of the myometrium [26]. Between 5-10 WG, interstitial EVTs migrate and invade both veins and lymphatics, but do not remodel their ECs [27], as well as invading uterine glands [28]. It remains to be investigated whether EVTs enter the maternal circulation and will trigger the maternal immune system to promote immune tolerance.

By 10 WG, open endings of maternal spiral arteries fill the placental lacunae (or intervillous space) with maternal blood, establishing utero-placental circulation [26].

The guidance for the migration towards the uterine spiral arteries happens via ephrin ligands on the EVTs that bind to ephrin type-B receptor 4 (EPHB2) on arteries and not to EPHB4 on veins [29]. EVT invasion is affected by the contact with the decidua, both maternal cells, and the ECM. Endovascular EVTs have been detected in decidual tissue “plugging” the spiral arteries at 8 WG, obstructing the spiral arteries while connecting with the intervillous space. Around 10 WG, the EVT “plug” is open, providing an open connection between the maternal spiral arteries and the intervillous space. The replacement of the VSMCs ensures that the spiral arteries cannot contract, as during the menstrual phase, resulting in detachment of the functional layer of the endometrium. By 15 WG, the myometrial arteries can also contain EVTs [26]. It remains unclear how the maternal veins are open into the intervillous space to allow blood flow into the maternal circulation.

The initial step of spiral arterial remodelling is likely regulated by immune cells that are localized in the vessels prior to EVT involvement [30]. The invasive EVTs, both interstitial and endovascular, then actively promote the dislodging of VSMC and ECs and changes in the ECM. Endovascular EVTs migrate against blood flow through the lumen and further interact with the ECs. Of note, the TBs form plugs in the first trimester, leading to less blood flow via the spiral arteries, suggesting that lower shear stress may favor TB migration [31]. The interstitial EVTs are likely in more contact with VSMCs [32]. However, it should be noted that interstitial EVTs have not been detected within the myometrium [29]. At this point, the EVTs undergo MEndT in that their expression profile is characterized by upregulation of VE-cadherin, platelet endothelial adhesion molecule-1 (PECAM-1, CD31), vascular cell adhesion molecule-1 (VCAM-1), α4 integrin, and αvβ3 integrin. These factors are all common vascular markers that are important for endothelial migration and adhesion. The maternal ECs and VSMCs present in the spiral arteries undergo apoptosis. This can result from the cell–cell attachment, but EVTs have also been shown to induce a direct apoptotic signaling [32]. Moreover, EVTs can induce caspase-dependent apoptosis in both ECs and VSMCs [33]. Furthermore, the ECM component elastin is degraded in the tunica intima and tunica adventitia, mediated by MMP-12, which is released by TBs and VSMCs [29]. Instead, the EVTs secrete fibrinoid components including fibronectin, collagen type IV, and laminin [32]. As a result of these steps, the spiral arteries become converted from narrow, high resistance vessels to wide, low resistance vessels that cannot contract, allowing adequate blood flow through the placenta to the fetus.

In preeclampsia (PE) these essential processes seem to fail, resulting in impaired placentation [13,34]. However, the molecular mechanisms are not understood. Emerging data demonstrate an important interplay between the transforming growth factor-beta (TGFβ) signaling pathway and other regulators in blood vessel formation; likely dysregulated in PE.

## 3. The TGFβ Family in Human Placental Development 

The TGFβ family consists of structurally related secreted cytokines, including TGFβ, activin, Nodal and bone morphogenetic proteins (BMP) that are implicated in the regulation of cell growth regulation, migration, deposition, and apoptosis of many tissues [35]. The TGFβ family has also been shown to play a huge role in placentation, however, with conflicting results (Table 1) [36]. Information on the role of the TGFβ family in nonhuman primates regarding placentation and implantation is limited, but is probably largely conserved with humans [9,10,37].

**Figure 4:**
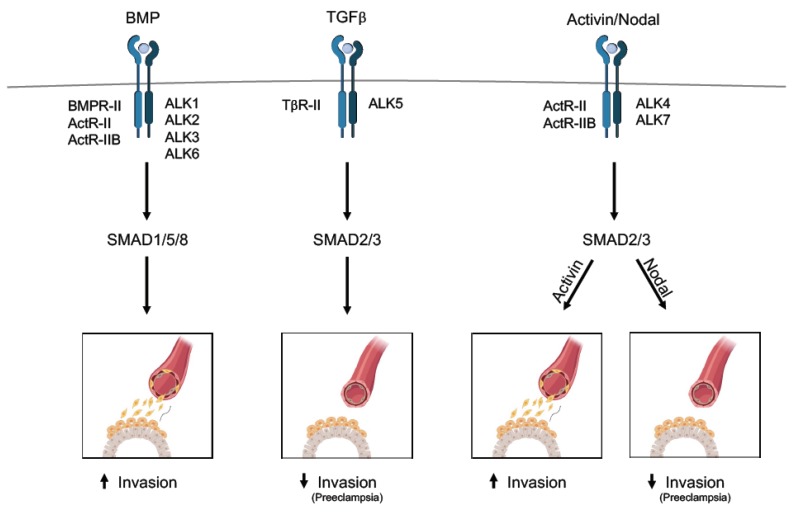
The proposed role of the TGFβ family in human placental development at the fetal–maternal interface. The TGFβ members signal by binding to receptor complexes type I (activin receptor-like kinase (ALK)) and type II receptor, depicted for each ligand. Upon receptor activation, the SMADs are phosphorylated and form complexes with SMAD4 to translocate into the nucleus, where they bind and initiate transcription of their target genes. The main members of the TGFβ family (TGFβ, bone morphogenetic protein (BMP), activin, and Nodal) have opposite roles in human placentation, either promoting or inhibiting trophoblast (TB) invasion. Hence, upregulation of TGFβ and Nodal signaling cascade is implicated in preeclampsia (PE). For clarification, Table 1 demonstrates the contradictory results reported on the role of TGFβ signaling in human placental development.

Signal transduction of the TGFβ family members is initiated by ligand binding to specific transmembrane type I and type II serine/threonine kinase receptors. TGFβ binds to the TGFβ type II (TβRII) receptor, which recruits and phosphorylates the TGFβ type I (TβRI) receptor, also known as activin receptor-like kinase (ALK), and leads to the formation of a receptor complex. In turn, the type I receptor passes the signal through phosphorylation of specific downstream mediators called receptor-regulated SMADs (R-SMADs) [35,51]. Subsequently, the phosphorylated R-SMADs associate and form complexes with SMAD4, also known as Co-SMAD, and accumulate in the nucleus where they regulate transcriptional activity of their target genes [52]. In general, TGFβ, activin, and Nodal lead to SMAD2/3 activation through specific type I and type II receptors, whereas BMPs lead to SMAD1/5/8 activation (Figure 4) [53].

### 3.1. TGFβ Cascade

TGFβ activates SMAD2/3 via the ALK5 type I receptor [53]. TGFβ signaling components are highly expressed in normal placenta but their levels decrease with gestational age [54,55]. Reports on the role of TGFβ in human TB invasion are contradictory (Table 1). Huang and colleagues observed that TGFβ1 promotes invasion of the human choriocarcinoma cell line JEG-3 via SMAD3 activation (but not SMAD2) and upregulation of the MMP2 and MMP9 [38]. The effect was abolished by the ALK4/5/7 inhibitor SB-431542. TGFβ1 promotes immortalized first trimester human CTB (HTR8/SVneo) differentiation by upregulation of cadherin-11. This upregulation is dependent on the TGFβ/SMAD2/3/SNAIL cascade [39]. Failure to abolish TGFβ3 expression in early pregnancy may lead to diminished TB invasion and in turn PE [56].

On the other hand, when investigating placental explants on matrigel, all the TGFβ isoforms hinder EVT invasion by inactivating proteases that are important for invasion [40]. Similar observations were reported earlier when using TGFβ1, which lead to blockage of invasive activity in HTR8/SVneo cells. This occurred via upregulation of plasminogen activator inhibitor (PAI-1) and tissue inhibitors of metalloproteases (TIMP) [41]. PAI-1 is a known TGFβ/ALK5/SMAD2/3 target [57] and inhibits urokinase plasminogen activator (uPA) activity, whereas TIMP is known to inhibit MMPs. Interestingly, Xu and colleagues demonstrated that LOX and LOXL2 are downregulated in preeclamptic placentae. Moreover, they identified the TGFβ1/SMAD3 cascade in mediating LOX and LOXL2 induced collagen production in the HTR8/SVneo TB cell line. If they inhibited the TGFβ1/SMAD3 activity in LOX and LOXL2 knockdown cells, they rescued the suppressed TB migration and invasion [23]. The TGFβ1/SMAD2/3-SMAD4 cascade can even upregulate cyclooxygenase-2 (COX-2), which in turn suppresses HTR8/SVneo TB cell invasion, suggesting TGFβ1/COX-2 as useful targets to treat placental disorders [58]. microRNA-218-5p (miR-218-5p) promotes TB invasion and endovascular EVT differentiation and accelerates the spiral artery remodelling process by targeting TGFβ2. Moreover, miR-218-5p was found to be reduced in PE placental tissues [59].

In summary, although debatable (Table 1), the majority of reports support the notion that TGFβ inhibits TB invasion at the fetal-maternal interface (Figure 4).

### 3.2. Activin and Nodal Cascade

The activin isoforms, activin A, activin B, and activin AB all act via the ALK4 and ALK7 type I receptors and are all expressed in the endometrium [60]. Activin A induces differentiation of first trimester CTBs into EVTs and EVT invasion [42,43]. The activin A-induced invasiveness of EVTs is mediated through the upregulation of SNAIL and activation of MMP2 and MMP9 [42,43]. Furthermore, the activins induce N-cadherin expression via SMAD2/3 to promote human TB invasion [61].

Nodal acts through the ALK7 type I receptor and activates SMAD2/3 signaling. Nodal inhibits TB differentiation, and promotes apoptosis and cell cycle arrest to inhibit TB proliferation [44,45]. Along the same line, Nodal also has an inhibitory effect on EVT invasion through downregulation of MMP2 and MMP9 [62].

Taken together, the reports suggest that activins promote TB differentiation into EVTs and EVT invasion, whereas Nodal is a potent regulator in placental development (Figure 4). What could the underlying mechanistic basis be behind those ligands that both activate SMAD2/3 and still elicit such different outcomes? Activin and Nodal may have different binding affinities for the activin type II A and B receptors and inversely modulate the activities of SMAD2 and SMAD3, which would result in an antagonistic effect, which has been shown in keratinocytes and tumor angiogenesis [63,64]. SMAD2 and SMAD3 can be arranged in a mixed trimeric complex in different combinations with or without SMAD4. This would result in a direct regulation of different transcriptional responses [65]. Hence, the antagonism may be based on the unique DNA binding MAD homology 1 (MH1) domain (SMAD2 has two additional short peptide inserts compared to SMAD3 [66]) and the different linker regions in SMAD2 and SMAD3. The linker region between the MH1 and MH2 domains could play a role in the opposite effect of Nodal and activin with regard to its phosphorylation status via the Ras/Mitogen-activated protein kinase (MAPK) pathway or phosphatases. Phosphorylation in the linker region could be different upon different Nodal/activin signaling instructions and inhibit C-terminal phosphorylation and in turn nuclear accumulation, attenuating the transcriptional activity of their target genes. Phosphatases that dephosphorylate the linker region are also likely to affect regulation of SMAD2 and SMAD3 activities.

### 3.3. BMP Cascade

BMPs comprise the largest group of the TGFβ family [67,68]. BMPs lead to activation of SMAD1/5/8 through BMP type I (ALK1, 2, 3, and 6) and type II receptors (Figure 4) [69]. BMPs can induce the expression of inhibitors of DNA binding (ID proteins) [70] that inhibit basic helix loop helix (bHLH) transcription factors, leading to angiogenic sprouting amongst other things [70]. BMP receptors are expressed in TBs, and placental endocrine hormones have been shown to either activate or suppress BMP signaling [71]. Gene ablation studies in mice and human vascular disease illustrate the importance of BMP signaling. Disruption in BMP signaling is reported to distort placental development and result in detrimental consequences for the embryo [72,73,74]. Mutations in endoglin (*ENG*) and activin receptor-like kinase 1 *(ACVRL1)* (encoding ALK1) have been linked to a human vascular disorder hereditary hemorrhagic telangiectasia (HHT1 and HHT2, respectively), often resulting in arteriovenous malformations (AVM) [75,76]. Another vascular disease caused by mutation in BMP receptor type II is hereditary pulmonary arterial hypertension (hPAH) [77].

BMP2 and activin A are expressed in the human endometrium and placenta. BMP2 induces human TB cell invasion by upregulating activin A and inhibin A. BMP2 initiates both canonical SMAD1/5/8 via the ALK3 receptor and non-canonical SMAD2/3 pathways [46]. The same group extended these studies and showed that BMP2 promotes TB cell invasion by upregulating N-cadherin via non-canonical ALK2/3/4-SMAD2/3-SMAD4 signaling [78]. They also revealed the role of BMP2 in promoting TB invasion and tube-like formation by ID1-mediated insulin-like growth factor binding protein-3 (IGFBP3) upregulation in primary and HTR8/SVneo cell cultures [47]. Indeed, it is becoming more evident that the TGFβ family uses combinatorial signaling via both SMAD cascades to induce EMT [79].

BMP4-induced signaling in combination with basic fibroblast growth factor (bFGF) is crucial for inducing EMT and mesodermal commitment of human human embryonic stem cells (hESCs) via SLUG and MSX2 [48]. MSX2 is also expressed in EVTs and induces TB invasion in human placenta [49]. The same investigators found that MSX2 expression was significantly lower in placental villi from PE patients compared to matched controls.

The BMP family members have been shown to play a big role in blood vessel formation [24,67]. BMP9 binds to the TGFβRI receptor ALK1 with high affinity in ECs, and the membrane bound co-receptor endoglin (ENG) induces this interaction [68]. The underlying mechanistic basis by which BMP9/ALK1 signaling can elicit an activating or inhibiting effect on angiogenesis is still unclear [75,80,81,82]. Different doses may induce distinct gene responses that are elicited at different thresholds of SMADs.

ENG is a homodimeric glycoprotein (180 KDa) and is constitutively expressed in the endothelium [83]. *Eng*-/- mouse embryos develop enlarged and weak vessels and lack smooth muscle cells surrounding them [84]. *Eng*-overexpressed ECs in mice dominate tip-cell position and home preferentially to arteries [85]. ENG is stably expressed in STBs, but only transient upregulation is observed in EVTs. Interestingly, loss of ENG promotes the invasion of HTR8/SVneo cells, and soluble ENG (sENG) has no effect on their invasiveness [86].

Soncin and colleagues performed comparative gene expression in the mouse/human placentae and identified BMP signaling components and related genes being upregulated in the first trimester in humans [50], and the human TSC model by Okae and colleagues [87] discussed later in the review is in line with these data.

In conclusion, most studies show that the BMP family members facilitate TB invasion at the fetal–maternal interface (Figure 4).

### 3.4. TGFβ Family-Associated Factors

ID proteins are BMP targets and function as dominant negative inhibitors of gene expression associated with cell differentiation. ID proteins dimerize with bHLH proteins and block their action. ID1 is strongly expressed in EVTs of human first trimester placenta compared to CTBs, suggesting that ID1 contributes to the differentiation of CTBs into EVTs [88]. BMP2 promotes TB cell invasion and endothelial-like tube formation by ID1-mediated IGFBP-3 upregulation, as demonstrated both in primary TBs and the HTR8/SVneo TB cell line [47]. ID2 downregulation is essential for CTB differentiation, but in cases of abnormal differentiation from TB in PE women, ID2 expression was constitutive [89]. Moreover, ID2 expression is downregulated during TGFβ-induced differentiation of the mouse TB progenitor cell line SM10 [90].

Epidermal growth factor-like domain 7 (EGFL7) is a secreted angiogenic factor that localizes to the ECM and can bind to αvβ3 and α5β1 integrins [91,92,93]. It is highly expressed during embryonic development in vascular and TB cells [94,95]. EGFL7 is often associated with the sprouting vessel and is important for regulating tubulogenesis [91], at least partly by modulating NOTCH signaling [91,96]. *EGFL*7 harbors the biologically active miR-126 within intron 7 in mammals [94,97]. miR-126 is an EC-specific miRNA [98,99,100] and is described as an important factor for vessel integrity [92,99,101]. Knocking down the *Egfl7* gene without affecting miR-126 demonstrates that *Egfl7* is essential during feto-placental vascularization and embryonic growth in the murine system [102]. Interestingly, EGFL7 secreted from EC in a murine system was found to be a negative regulator of vascular elastogenesis; EGFL7 acts through a direct interaction with the catalytic domain of the lysyl oxidase that hinders lysyl oxidases to cross link elastin [103]. Junus and colleagues investigated differential gene expression in placentae between early and late onset PE and identified *ACVRL1* and *EGFL7* genes in early onset disease [104]. In a recent paper, BMP9 was demonstrated to promote, via its receptor ALK1 and upregulation of EGFL7, sprouting angiogenesis of hESC-derived endothelial cells [105].

Endothelin-1 (ET-1) is a mitogen and potent vasoconstrictor. Both TGFβ and BMP9 upregulate ET-1 production in human lung blood microvascular ECs, which may contribute to the pathogenesis of PAH [106]. ET-1 expression is also localized in the placenta and is highly induced in PE, suggesting a similar cause of PE as for PAH [107].

## 4. Preeclampsia 

The pregnancy syndrome preeclampsia (PE) affects approximately 2-8% of all pregnancies and is the leading cause of maternal and fetal mortality worldwide [108]. PE is a common yet unresolved complication that is characterized by elevated blood pressure (hypertension) and an excess of proteins in urine (proteinuria) after 20 WG that can affect the kidney, liver, and brain [109]. The placenta is both necessary and sufficient to cause the disease, and delivery of the placenta is the only cure [34]. Imbalance of pro- and anti-angiogenic factors has been suggested, resulting in maternal endothelial dysfunction and increased vascular permeability [110]. Due to the similarities in fetal–maternal interface, preeclampsia may also be observed in great apes [10,12], including gorillas [111]. One major problem in investigating causes and/or treatments of preeclampsia is the lack of suitable non-hominidae animal models. Rodent, sheep, rabbit, rhesus monkey, and baboon have been used to mimic preeclampsia. However, those animal models only develop hypertension and not other parameters associated with PE. Hence, although interesting, those are of limited use [9,112].

Soluble Fms-like tyrosine kinase 1 (sFLT1) and soluble ENG (sENG) are both elevated in the serum of PE patients, weeks before clinical manifestations of the disease, and have therefore received much attention. sFLT1 acts as a decoy for vascular endothelial growth factor (VEGF) and placental-derived growth factor (PlGF) [83]. The sFLT isoform is the product of the FLT-1 splice variant [113]. sENG is formed by cleavage of membrane bound ENG by MMP14 [114]. After it was revealed that ENG has very high affinity to BMP9 and BMP10 (but only minor to TGFβ1 and TGFβ3 ligands) the BMP9 and BMP10 have been thought to be trapped by sENG [83,115], suggesting BMP proteins as potential biomarkers for PE. The levels of BMP submembers have not been investigated extensively in blood from PE patients. There is no difference in circulating BMP9 levels from blood between PE patients and controls. Some reports show that sENG is significantly higher in plasma from PE patients, but logistic regression analysis describes that sENG levels are independently associated with the development of PE [116]. A complex of sENG (monomer)/BMP9 has been detected in both PE patients and a control group, being slightly higher in the PE group [117]. Lawera and colleagues observed that binding of sENG to BMP9 does not inhibit BMP9 signaling [117]. Moreover, the increased circulating sENG might rather direct BMP9 signaling via cell-surface ENG.

Expression analysis in placentae from PE women has shown reduced expression of members of the BMP signaling pathway (*ALK1, ID1,* and *EGFL7)* [95,104,118]. Together with the known sFLT1 and sENG biomarkers of PE, the molecular mechanism is narrowed down to the BMP signaling pathway and possible cross-talk with VEGF and NOTCH signaling.

Studies on TGFβ isoforms in PE are contradictory [37]. TGFβ1 and TGFβ2 are elevated in the plasma of PE patients [36]. Reduction of TGFβ3 causes TBs to become less invasive, leading to PE [56]. Moreover, TGFβ3 is highly expressed in placentae from PE patients, and PE explants exhibit failure of invasion in vitro [56]. On the other hand, it has been demonstrated that none of the TGFβ isoforms are expressed in villous TBs, but are all present in the placental bed (although TGFβ3 was low). Only TGFβ2 was found to be present in EVTs [119]. Moreover, some studies have shown no difference in the levels of TGFβ1 in serum of PE vs. normal pregnancies [116]. Applying a bioinformatics approach combined with cell experiments, Wu and colleagues demonstrated that TGFβ signaling cascade was critical for the development of preeclampsia [120]. These inconsistencies may be due to different cell types/systems studied or differences in methodology, such as antibody specificity.

Alahari and colleagues sought to find whether TGFβ3 was involved in epigenetic regulation of von Hippel Lindau tumor suppressor gene (*VHL*) in the human placenta [121], and discovered that TGFβ downregulated *VHL* mRNA. Moreover, in line with TGFβ increase in early onset PE, E2F4-*VHL* association was enhanced upon TGFβ3 stimulation, suggesting *VHL* transcriptional inhibition.

The expression of Nodal and ALK7 in placentae was detected in villous and extravillous TBs in early gestation. Nodal and ALK7 levels were strongly increased in placentae from PE. Overexpression of Nodal or constitutively active ALK7 decreased TB cell migration and invasion [62]. Activin A levels were found to be elevated in serum from PE patients compared to normal pregnancies [122,123,124]. A polymorphism in the *ACVR2A* promoter region (rs1424954) was significantly associated with PE and caused a decrease in *ACVR2A* expression [125]. This PE susceptibility allele causes activin A effects on Nodal expression to reduce in TBs under physiological activin A levels, meaning that it is protective, whereas under pathologic activin A levels, the expression of Nodal still increases due to the lack of ACVR2B downregulation. This results in reduced TB invasion, as seen in PE placentae [125].

In summary, the role of the TGFβ family components in human placental development and PE is still highly contradictory and awaits further elucidation. One reason could be due to different isolation methods from the placentae that ends in different TB subtypes.

## 5. Stem Cells for Human Placental Development and Organoids

Single-cell sequencing technology has revealed similarities and, most importantly, differences in gene expression between mouse and human blastocyst embryos, including the trophoectoderm (TE) lineage [126,127], helping to explain why it has been so challenging to derive and maintain human TSCs. As an example, human blastocyst TE cells showed species-specific expression of claudin 10 (*CLDN10*) and placenta associated 8 (*PLAC8*), whereas mouse blastocyst TE cells showed species-specific expression of E74-like factor 5 (*Elf5*) and eomesodermin (*Eomes*) [126,128]. In addition, although the human blastocyst stage is now rather well characterized, it remains challenging to understand the human TE developmental trajectory during the period of implantation and gastrulation [128]. However, pioneer studies are revealing the single cell signature of human blastocyst outgrowths cultured from 6 to 14 days post-fertilization (dpf) regarding transcriptomics and methylomics [129]. By contrast, studies in mouse are highlighting the complex developmental dynamics of the mouse TE lineage during implantation and gastrulation [130]. The comparative analysis of these datasets will surely contribute to a better understanding of the transcriptional factors, signaling pathways, and extracellular environment that may be necessary to maintain the population of human TE cells in culture as bona fide TSCs [131].

Recent attempts to derive human TSC from primary cells, such as TE cells isolated from 5–6 dpf blastocysts and from (double ITGA6/KRT7-positive) CTBs isolated from chorionic villi of 6–9 WG placentae, have resulted in cell lines with the potential to self-renew and differentiate into cells with characteristics of EVTs (major histocompatibility complex, Class I, G (HLA-G) and chorionic gonadotropin subunit beta (CGB)) or STBs (CGB and syndecan 1 (SDC1)) [87]. Important for the derivation and maintenance of human TSCs was the activation of the WNT pathway (CHIR99021) and epidermal growth factor (EGF) pathway, while inhibiting the TGFβ pathway (A83-01 and SB431542), histone deacetylases (VPA), and Rho-associated protein kinases (Y27632). It remains to be determined how similar these in vitro differentiated cell types are to the in vivo counterparts, and how these can be implemented in disease models to understand implantation and placentation.

In addition to TSCs derived from human TE-derived primary material, differentiation protocols that include exogeneous treatment with BMP4 applied to human pluripotent stem cells (PSCs) seemed to result in cells with TE characteristics [128,132]. Some studies reported, for example, expression of CDX2, but also of ELF5 and EOMES, both known to be absent from human TE cells. Although still unclear, these cells may in fact represent extraembryonic mesoderm-like cells, instead of TE-like cells [128]. Converting mouse and human PSCs into a novel “extended” pluripotency state by treatment with leukemia inhibitory factor (LIF), CHIR99021, (S)-(+)-dimethindene maleate, (DiM) and minocycline hydrochloride (MiH), Yang and colleagues demonstrated that these PSCs had the ability to integrate into the placenta of chimeric mice [133], although the specific cell lineage (TE or extraembryonic mesoderm) in the placenta remains under debate. Exciting recent results from Kime and colleagues using mouse PSC have shown that the endogenous retrovirus Mervl gene activation (characteristic of a 2C pluripotency stage) represents a pluripotent state of cells that can self-organize into cavity-filled three-dimensional (3D) structures that morphologically resemble blastocyst-like cysts, containing both inner cell mass (ICM)-like cells and TE-like cells [134]. Although these blastocyst-like cysts are able to induce a decidualization reaction in the uterus of pseudo-pregnant female mice, they degenerate a couple of days after transfer, failing to develop even to the egg-cylinder. The field of synthetic embryology is only recently emerging, and if these initial steps can be translated from mouse to human, this could allow the development of models of implantation based on (pluripotent) stem cells. Importantly, it is forbidden to culture human embryos beyond 14dpf, and the ethical and legal status of these so-called artificial embryos should be debated.

Pioneer work on placental spheroids from, for example, immortalized EVTs, trophoblast Jar cell line, and the choriocarcinoma cell line BeWo have been used as 3D models to study aspects of human placental development in vitro [135,136,137] and, when co-cultured with human immortalized endometrial stromal cell lines, have served as a model for TB invasiveness [138,139]. Moreover, attempts have been performed to develop 3D models for human endometrial cells that could also be used to investigate endometrial cancer [140,141,142].

In addition, the development of 3D organoid models using human placental tissue and endometrial tissue has also been attempted and shows promising clinical relevance in the study of pregnancy-related diseases [13,131]. Organoids are small aggregates of cells that originate from a single (or very few) cells, either pluripotent stem cells or organ progenitors, and that can grow and self-organize into a 3D structure that recapitulates a specific function of a specific organ (of origin). They contain several cell lineages that segregate into cellular compartments, mimicking a mini-organ [143]. As organoids can be generated from patient-specific induced pluripotent stem cells (iPSCs), they can be of value for patient-specific disease modelling and drug discovery, leading to personalized medicine.

Placental organoids, of which functionality is the ability to express or produce human chorionic gonadotropin, have been derived from CTBs purified from 6–7 WG placentas, but not from 10–12 WG placentas [131,144]. This was achieved by using a cocktail containing activators of the WNT pathway (prostaglandin E2, CHIR99021, and R-spondin), HGF, and EGF pathway (HGF, EGF), while inhibiting the TGFβ pathway (A83-01) and BMP pathway (Noggin), being grown on Matrigel. These formed compact cell aggregates containing multiple layers of CTBs instead of a single epithelial layer (as found in the chorionic villi) and, as expected, were devoid of (vimentin-positive) mesenchymal cells and hence of developing vasculature. The CTBs in the 3D organoids could differentiate into cells with properties of STBs or EVT, similar to the 2D culture-differentiated derivatives of hTSCs [87]. Although these models may be useful to understand differentiation to different TE-derived cells, it will be desirable to integrate them with mesenchymal cells in order to allow vasculature development, or with endometrial cells to study the fetal–maternal interface.

Organoids from human (non-pregnant) endometrium, able to undergo long term expansion, have also been developed [145,146]. Those cystic-organoids seem to be able to mimic some aspects of the endometrial glandular epithelium, responding to sequential hormonal changes in estrogen and progesterone as during the menstrual cycle. After estrogen treatment, the epithelium of endometrial organoids showed pseudostratified organization, resembling the proliferative phase. After progesterone treatment, the epithelium became columnar and showed increase folding as in the secretory phase. Hormone withdrawal resulted in increased apoptosis as in the menstrual phase. These organoids can be isolated from patients undergoing laparoscopy and can be cultured in Matrigel in medium containing insulin, transferrin and selenium (ITS), estrogen (E2), activators of the WNT pathway (R-spondin), and FGF and EGF pathway (FGF10, EGF), while inhibiting the TGFβ pathway (A83-01) and BMP pathway (Noggin), and being grown on Matrigel. In addition, when (glandular) endometrial organoids were stimulated with pregnancy-associated hormones (chorionic gonadotropin, placental lactogen, prolactin), they started producing components such as progestagen-associated endometrial protein (PAEP), of the so-called “uterine milk”, characteristic of gestational endometrium [146]. Endometrial organoids have already been isolated from patients with a broad spectrum of endometrial pathologies, such as endometriosis, endometrial hyperplasia, Lynch syndrome, and endometrial high- and low-grade cancer, and used as a disease model for drug discovery and better understanding of the pathologies [146,147].

Although these models are promising for several applications, organoid models of the placenta and endometrium are limited in their functionality due to the lack of the mesenchymal compartment, which contains the vasculature, pivotal for the fetal–maternal interface, allowing nutrient and gas exchange, as well as immune tolerance. In this regard, there is room to improve, perhaps incorporating aspects of organs-on-chip in a microfluidic setup [148], which could include the missing vascular compartment. Another interesting development will be to use microfluidics to multiplex several organoid systems representing different body organs, such as the Evatar, which constitutes a multiplex of female reproductive tract organs, including the ovary, fallopian tube, uterus, cervix, and liver to study endocrine loops and drug discovery [149].

## 6. Concluding Remarks

Taken together, there is still great controversy about the TGFβ signal transduction machinery involved in human placental development and the fetal–maternal interface, whether touching upon migration, invasion, or spiral arterial remodeling. However, on the basis of the numerous reports on the importance of TGFβ signaling components, there must be a consensus that this family of growth factors has a pivotal role in human placental development and establishing the fetal–maternal interface. There is a current burst of new technologies related to human TSCs and placental/endometrial organoids, leading to models that have been developed to understand their differentiation into different subtypes of TBs and the crosstalk with the endometrium. Moreover, the use of single cell technologies to robustly identify the molecular signature and developmental trajectory of each population of cells involved in the establishment of the fetal–maternal interface will pave the way for a better understanding of each step during human placentation and uterine decidualization. Finally, the generation of fetal-maternal interface models will be fundamental to unravel causes of pregnancy-related complications, but also to develop (personalized) treatments leading to increased reproductive success.

## Figures and Tables

**Table 1:** The role of the TGFβ family in human placental development. FT: First trimester, JEG-3: Human choriocarcinoma cell line, HTR8/SVneo: Immortalized first trimester human CTBs, Rcho-1: Rat choriocarcinoma cell line, JAR: Human choriocarcinoma cell line.

TGFβ FACTOR	ROLE	CELL LINE/SYSTEM	REFERNCES
TGFβ1	- ↑ Promotes invasion - ↑ Promotes FT CTBs differentiation - ↓ Inhibits EVT invasion - ↓ Inhibits invasion	- JEG-3 - HTR8/SVneo - Primary EVTs - HTR8/SVneo	[38] [39] [40] [41]
TGFβ2	- ↓ Inhibits EVT invasion	- Primary EVTs	[40]
TGFβ3	- ↓ Inhibits EVT invasion	- Primary EVTs	[40]
ACTIVIN A	- ↑ Induces FT CTBs → EVTs - ↑ Promotes EVT invasion	- FT chorionic villi explants - Primary FT CTBs	[42] [43]
NODAL	- ↓ Inhibits TB differentiation - ↑ Promotes apoptosis - ↓ Inhibits proliferation	- Rcho-1, TSC - JEG-3 - HTR8/SVneo, JEG-3, JAR	[44] [45] [45]
BMP2	- ↑ Promotes EVT invasion - ↑ Promotes tube-like formation	- FT placental explants - FT placental explants, HTR8/SVneo	[46] [47]
BMP4	- ↑ Induces EVT invasion via MSX2	- HTR8/SVneo, JEG-3, JAR	[48,49]
ENG	- Loss of ENG promotes invasion	- HTR8/SVneo	[50]
